# Supplementary material for: MetaRibo-Seq measures translation in microbiomes
Source: Nat Commun. 2020 Jun 29;11:3268. doi: 10.1038/s41467-020-17081-z (PMC7324362; doi:10.1038/s41467-020-17081-z)
Supplement: Supplementary file 10 — Supplementary Data 7 [file 41467_2020_17081_MOESM10_ESM.zip › File2/Confidence_VeryHigh_Taxonomy/261679_out.krona.html]

Javascript must be enabled to view this page.

members
magnitude
magnitudeUnassigned
count
unassigned
taxon
rank

261679\_out

14

2
superkingdom
13

1239
phylum
13

186801
class
13

13
186802
order

12
31979
family

12
genus
1485

12
species

SRS013965\_contig\_number\_35258SRS014235\_contig\_number\_12253SRS015190\_contig\_number\_contig-100\_1809.43437SRS022137\_contig\_number\_19762SRS042628\_contig\_number\_contig-100\_24272.95155SRS043411\_contig\_number\_contig-100\_1080.47344SRS043768\_contig\_number\_contig-100\_3260.155587SRS063985\_contig\_number\_27768SRS077335\_contig\_number\_contig-100\_3454.33955SRS104311\_contig\_number\_30670SRS143598\_contig\_number\_5910SRS148159\_contig\_number\_14050
1262789

1
family
186803

species

SRS104400\_contig\_number\_30845
1952130
1


SRS148196\_contig\_number\_contig-100\_52084.52084
1
